# Microwave-assisted conversion of palm kernel shell biomass waste to photoluminescent carbon dots

**DOI:** 10.1038/s41598-020-78322-1

**Published:** 2020-12-03

**Authors:** Wei Lun Ang, Cheldclos A. L. Boon Mee, Nonni Soraya Sambudi, Abdul Wahab Mohammad, Choe Peng Leo, Ebrahim Mahmoudi, Muneer Ba-Abbad, Abdelbaki Benamor

**Affiliations:** 1 Department of Chemical and Process Engineering, Faculty of Engineering and Built Environment, Universiti Kebangsaan Malaysia, 43600 Bangi, Selangor Darul Ehsan Malaysia; 2 Centre for Sustainable Process Technology (CESPRO), Faculty of Engineering and Built Environment, Universiti Kebangsaan Malaysia, 43600 Bangi, Selangor Darul Ehsan Malaysia; 3 Chemical Engineering Department, Universiti Teknologi PETRONAS, 32610 Seri Iskandar, Perak Darul Ridzuan Malaysia; 4 Center for Advanced Integrated Membrane System (AIMS), Universiti Teknologi PETRONAS, 32610 Seri Iskandar, Perak Darul Ridzuan Malaysia; 5 School of Chemical Engineering, Engineering Campus, Universiti Sains Malaysia, 14300 Nibong Tebal, Penang Malaysia; 6 Gas Processing Centre, Qatar University, P.O. Box 2713, Doha, Qatar

**Keywords:** Chemical engineering, Environmental chemistry

## Abstract

In the present work, palm kernel shell (PKS) biomass waste has been used as a low-cost and easily available precursor to prepare carbon dots (CDs) via microwave irradiation method. The impacts of the reacting medium: water and diethylene glycol (DEG), and irradiation period, as well as the presence of chitosan on the CDs properties, have been investigated. The synthesized CDs were characterized by several physical and optical analyses. The performance of the CDs in terms of bacteria cell imaging and copper (II) ions sensing and removal were also explored. All the CDs possessed a size of 6–7 nm in diameter and the presence of hydroxyl and alkene functional groups indicated the successful transformation of PKS into CDs with carbon core consisting of C = C elementary unit. The highest quantum yield (44.0%) obtained was from the CDs synthesised with DEG as the reacting medium at irradiation period of 1 min. It was postulated that the high boiling point of DEG resulted in a complete carbonisation of PKS into CDs. Subsequently, the absorbance intensity and photoluminescence intensity were also much higher compared to other precursor formulation. All the CDs fluoresced in the bacteria culture, and fluorescence quenching occurred in the presence of heavy metal ions. These showed the potential of CDs synthesised from PKS could be used for cellular imaging and detection as well as removal of heavy metal ions.

## Introduction

Recently, due to its unique characteristics such as tunable photoluminescence property, good biocompatibility, and photostability, carbon dots (CDs) has emerged as another popular member of carbon group that has obtained significant attention from the researchers^1–4^. CDs are discrete, multicolour photoluminescence quasi-spherical nanoparticles (with a diameter of less than 10 nm) that consist of sp^2^/sp^3^ hybridised carbon atoms and multiple functional groups such as carboxyl, hydroxyl, and amino groups^5–7^. Upon excitation by UV-radiation, CDs can emit bright fluorescence^8^. The superior electronic properties as electron donors and acceptors entrust CDs with applications in sensors, catalysis and optronics^9^. The photoluminescence property of CDs has found great potential in various analytical applications such as for the detection of pollutants (e.g. heavy metals, pesticides, and persistent organic pollutants) and vitamins^10–16^. Thanks to the outstanding biocompatibility and low toxicity, CDs have also emerged as the more preferable and feasible materials in biomedical fields (such as biosensing, delivery of biomolecule/drug, and bioimaging) as compared to the conventional quantum dots (metal-based semiconductor) that lead to biosafety and health concerns^17–20^.

The synthesis of CDs can be generally classified into two approaches, which are top-down and bottom-up methods using chemical, electrochemical or physical techniques. The former approach involves techniques such as laser ablation, arc discharge, and electrochemical techniques, whereas the bottom-up approach comprises techniques like hydrothermal treatment, solvothermal treatment, chemical treatment, plasma treatment, and microwave synthesis^1,21^. Among the aforementioned synthesis techniques, microwave-assisted synthesis is gaining more attention since it is considered as a green and economical approach that provides fast heating and easy to scale up without suffering thermal gradient effects^20,22^. Several articles on the successful synthesis of CDs using microwave-assisted synthesis have been reported^20,22–24^. For instance, using citric acid, urea, and thiourea as the raw materials, CDs with the capability in detecting mercury and iodide compounds in aqueous solution have been successfully fabricated through microwave-assisted method^23^. In another case, eggshell membrane ashes which were converted to CDs reportedly possessing photocatalytic degradation capability in reducing the methylene blue contaminants^24^.

One of the important factors that contribute to the rise of CDs is due to its easily available and low-cost carbon precursors, ranging from organic molecules, agricultural and natural products, to even the biomass wastes^1, 23,25–27^. Recycle and reuse of the waste with carbon-rich as precursors to prepare the CDs appears to be a good effort along with the global concern in developing a sustainable community through waste minimisation. Furthermore, the wastes are renewable resources and cheap in terms of cost^28^. Several biomass wastes have been explored by the researchers as the carbon source for the synthesis of CDs, for instance sago waste, sugarcane molasses, and food waste^7,27,28^. Huang et al. (2017) successfully converted sugarcane molasses (biomass waste) into CDs that emitted blue photoluminescence with a quantum yield of approximately 5.8%^7^. The synthesised CDs exhibited great photostability and strong resistance to photo-bleaching with no obvious change in fluorescence intensity upon continuous exposure to UV light. Furthermore, the CDs demonstrated excellent biocompatibility with living cell (bioimaging) and capable of detecting Fe^3+^ and sunset yellow (synthetic organic food dye) through fluorescence quenching. These showed that CDs synthesised from natural compounds or biomass are as competent as CDs fabricated from other sources. However, the simple synthesis (using water as the reaction medium) of CDs from biomass wastes tend to have low quantum yield due to incomplete carbonisation and the precursors lack functional groups (sulfur or nitrogen atom) for better luminescence property^20,29–34^.

There are several ways to improve the quantum yield of CDs from biomass wastes, such as surface passivation, heteroatom doping, and reaction conditions^30^. Of these, controlling the reaction conditions and manipulating the carbon precursors composition is of particular interest. For instance, Wee et al. (2013) observed that the rise in temperature had improved the quantum yield of the CDs^35^. This could be attributed to better carbonisation conversion rate from carbon precursors (bovine serum albumin) to CDs at higher temperature (room temperature vs 50 °C). The motion of the precursors was excited at higher temperature, and subsequently the efficiency of the carbonisation process was increased too. However, carbonisation efficiency was hindered when water was used as the reaction medium since the temperature could not rise high due to low boiling point of water. Hence, by replacing water with a high boiling point solvent such as diethylene glycol (DEG), the yield of CDs could be improved^20^. In another case, substances with amino groups (-NH_2_) such as chitosan have been included as one of the precursors to boost the synthetic yield of CDs. The amino-functional groups which containing nitrogen improved the quantum yield due to its great fluorescence property^9,36^. Though a great number of studies have successfully improved the quantum yield and fluorescence property of CDs through heteroatom doping, majority of the studies were conducted on simple carbon precursors such as citric acid, polyacrylic acid, and glycine^23,37,38^. Similar study on natural carbon precursor has remained rare. On top of that, the reaction conditions, especially the reacting medium on the conversion of natural carbon precursor to CDs and effect on the quantum yield has never been properly investigated.

Hence, in this study, the influence of the reaction medium (DEG) and amino group compound (chitosan) on the microwave-irradiation synthesis of CDs by using natural carbon precursor—palm kernel shell (PKS), was investigated. PKS is a solid biomass waste from the palm oil industry. The palm oil industry is the most important agricultural sector in Malaysia and associated with its role in contributing to the economic growth, a significant amount of PKS (> 2.4 million tons yielded per annum) has been generated^39^. This solid waste has low economic value and is normally disposed as landfill or used as a low-rank fuel to be burned for heat/steam generation. Converting this low-cost solid waste to useful CDs will help to resolve the issues of waste management and at the same time supporting sustainable development. The potential of the synthesised CDs was further explored for cellular imaging, detection and removal of heavy metal ions.

## Methodology

### Chemicals and materials

Palm kernel shell (PKS) as the precursor for CDs synthesis was obtained from Tennamaram palm oil mill located at Bestari Jaya, Selangor, Malaysia. Diethylene glycol (DEG) was purchased from Labchem Sdn. Bhd. (Malaysia). Chitosan and quinine sulfate was purchased from Sigma-Aldrich (M) Sdn. Bhd. (Malaysia). Ultrapure water (UPW) (~ 18.2 MΩ m, 25 °C) obtained from Sartorius Arium PRO (Fisher Scientific, USA) water purification system was used throughout the experiment. Copper (II) sulfate and phosphate buffer solutions (PBS) were purchased from Merck, Malaysia.

### Synthesis of CDs

The PKS was ground into a powder and sieved with a sieve size of 0.2 mm to obtain fine powder for CDs synthesis. Firstly, an appropriate mass of PKS powder was weighed and then mixed with the three solutions: UPW (Set A); UPW with chitosan (amount of chitosan was the same as PKS) (Set B); and DEG (Set C), separately, to form a mixture with PKS concentration of 10 g/L in a Duran bottle. DEG was included as the reaction medium in this study to investigate its influence on the CDs as it has higher boiling point that might facilitate a more complete carbonization process^20^. The mixtures were homogenised by sonicating the solutions for 30 min. The samples were then heated in the microwave oven (Midea MM720CXM, Malaysia) at medium heating power (385 W) and heating period ranging from 1 to 5 min. Table 1 shows the labelling for each set with varied heating periods and solution formulation. The resulting product was subjected to cooling process to room temperature after the carbonisation process. The mixture was then filtered with 0.45 μm membrane filter, and the filtrate was centrifuged at 10,000 rpm for 20 min to remove the bulk particles. Finally, the supernatant was filtered again with syringe filter (0.22 μm) and then collected for further analysis and application testing.

**Table 1:** Labelling for each set with varied heating periods and solution formulation.

Parameter	Heating period (min)				
	1	2	3	4	5
A	A1	A2	A3	A4	A5
B	B1	B2	B3	B4	B5
C	C1	C2	C3	C4	C5

### Characterisation of CDs

Transmission Electron Microscope (TEM) (Thermo Fisher, Talos 120C, United States) was used to obtain the high-resolution images of the synthesised CDs. The functional groups that present on the PKS and CDs were characterised by using Fourier Transformed Infrared (FTIR) (Nicolet, 6700, United States). UV–visible absorption spectra of the CDs were determined by using Lambda 950 UV–visible spectrophotometer (Perkin Elmer, United States). Photoluminescence spectra of the CDs was determined via FLS920 fluorescence spectrophotometer (Edinburgh Instrument, United Kingdom). The Raman spectra were obtained using Raman spectrophotometer equipped with a laser source emitting a wavelength of 532.230 nm (WITec, Model: Alpha 300R, USA). X-ray diffraction (XRD) spectra were generated via X-ray diffractometer (Bruker D8 Advance, Germany). The sample preparation for XRD analysis involved the dropping and drying of CDs on a glass slide for several cycles. The surface chemical composition of the CDs was analyzed by X-ray photoelectron spectroscopy (XPS) (Ultra DLD XPS Kratos, Manchester, UK) equipped with a monochromatic Al Kα radiation source (1486.6 eV).

### Measurement of quantum yield

The quantum yield of the prepared CDs was calculated according to the established method by using the following equation:

1 $$\Phi _{s} = \Phi _{{\text{R}}} \times \frac{{\text{I}}}{{{\text{I}}_{{\text{R}}} }} \times \frac{{{\text{A}}_{{\text{R}}} }}{{\text{A}}} \times \frac{{\upeta ^{2} }}{{\upeta _{{\text{R}}}^{2} }}$$

where η, *A*, Φ, and *I* are the refractive index of the solvent, absorbance at the excitation wavelength, quantum yield, and integrated fluorescence intensity, respectively^22^. The subscript R refers to the reference fluorophore of known quantum yield while the terms with the subscript s indicate the synthesized CDs. In this study, quinine sulfate was used as the reference fluorophore. To prepare the solution for measurement, quinine sulfate (Φ_R_ = 0.54) was dissolved in 0.1 M H_2_SO_4_ (η = 1.33) and CDs were dissolved in deionized water (η = 1.33). Absorbance in the 1 cm quartz cuvette was kept below 0.10 at an excitation wavelength of 370 nm to minimize re-absorption effects^40^.

### Cellular imaging

The culture of *Escherichia coli* (*E. coli*) and Bacillus subtilis bacterial cells were carried out in a sterile environment by using the equipment that had been sterilized in autoclave. The bacterial cells were cultured in sterile nutrient agar. The colony of the bacterial cells was taken from the agar plates and then incubated in 10 mL of nutrient broth. The bacterial cultures were grown overnight at 37 °C in a shaking incubator. On the next day, 150 µL of CDs solution was diluted to 600 µL with ultrapure water for the reaction with 400 µL of bacterial cells for 60 min at 37 °C with gentle shaking. To remove the supernatant, the mixture solutions were then centrifuged at 10,000 rpm for 10 min. The residue was then washed with phosphate-buffered saline (PBS) buffer and suspended in PBS buffer^41^. Lastly, the cells were observed under inverted fluorescence microscope (Nikon TI-E, Japan) after 20 µL of culture has been added to the glass slide^40^.

### Detection of heavy metal ions

Heavy metal solution (copper (II) sulfate solution) with different concentrations (0125, 0.25, and 0.5 M) were used to evaluate the heavy metal ions detection capability of the synthesized CDs. 5 mL of CDs solution and 5 mL of copper (II) sulfate solution with different concentrations were mixed and the effectiveness of metal ions sensing was evaluated through the quenching of photoluminescence spectrum of CDs upon excitation at 370 nm.

### Removal of heavy metal ions

Removal of heavy metal ions was evaluated by adding different doses of CDs into the synthetic solution (0.5 M of copper (II) sulfate solution). In the first place, 6 mL of 0.5 M copper (II) sulfate solution was mixed with 1 mL of CDs, and the removal efficiencies were recorded at 10, 20, 30, 40, and 50 min. Next, different volumes of CDs (0.5, 1.0, 1.5, 2.0, and 2.5 mL) were added to 6 mL of 0.5 M of copper (II) sulfate solution for investigating the influence of CDs dosage on the removal efficiencies. Performance of the removal of copper (II) ions was evaluated by measuring conductivity of the solution using a HI 2550 Benchtop Meter (Hanna, USA).

## Results and discussion

### Determination of optimum synthesis conditions

This study was started with the determination of optimum microwave irradiation synthesis conditions. As one of the key factor, the heating period played an important role in determining photoluminescence intensity of CDs^20^. The heating period has been studied to obtain optimum synthesis condition in preparing CDs with the highest quantum yield from different reaction medium. As shown in Table 2, the highest quantum yield for CDs synthesized from set A (26.3%), set B (26.3%), and set C (44.0%) were obtained at the heating duration of 4 min (A4), 5 min (B5), and 1 min (C1), respectively. The relatively shorter optimal heating duration and higher quantum yield for set C indicate the contribution from the DEG reacting medium where further explanation would be provided in the following section. Unexpectedly, the quantum yield of set B CDs did not differ from set A, indicating that the chitosan did not react with the PKS to form CDs with amino functional groups as observed in other studies^33^. The CDs samples (A4, B5, and C1) were then characterized and analyzed to understand the factors contributing to this variation in quantum yield.

**Table 2:** Quantum yield of CDs at different synthesis conditions.

	Parameter	Heating period (min)				
		1	2	3	4	5
Quantum Yield (%)	A	12.1	19.3	23.6	26.3	15.5
	B	13.0	14.3	18.6	22.9	26.3
	C	44.0	15.8	6.3	2.3	3.9

### Characterization of CDs

**Figure 1:**
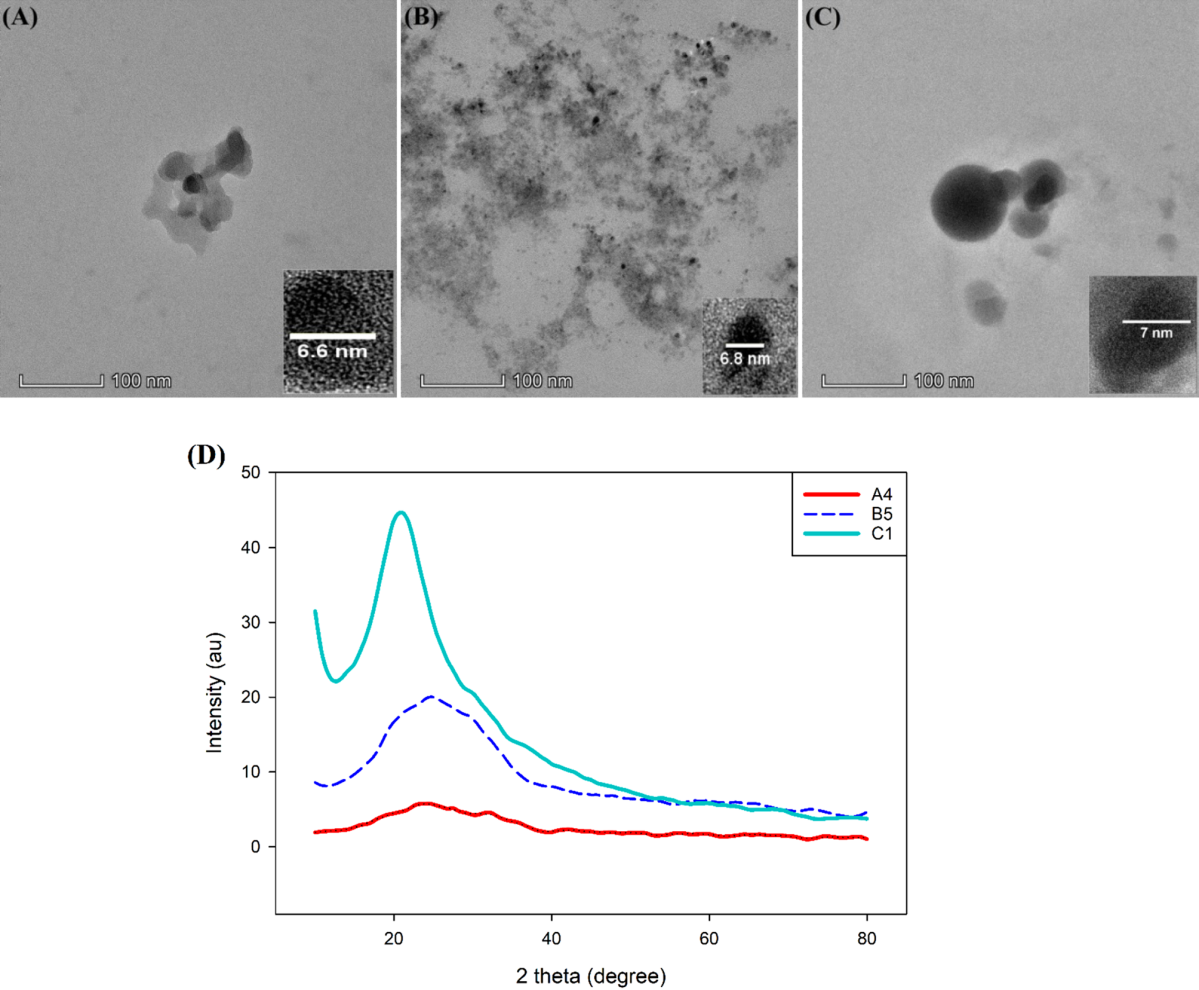
TEM image for sample (**A**) A4 (**B**) B5 and (**C**) C1 (scale bar is 100 nm) and (**D**) XRD spectrum of CDs.

The size and morphology of synthesised CDs under optimal synthesis conditions were observed by TEM. Figure 1A–C shows that the synthesized CDs of sample A4, B5 and C1 were quasi-spherical nanoparticles with and average diameter of 6.60 nm, 6.80 nm and 7.00 nm, respectively. The CDs were postulated to be amorphous carbon particles due to the absence of crystal lattices as revealed by the TEM images. This postulation was supported by the XRD of CDs as shown in Fig. 1D. The peak positioned at about 2θ = 25° which is in accordance with other studies referring it as an indication of amorphous carbon phase^42–44^.

**Figure 2:**
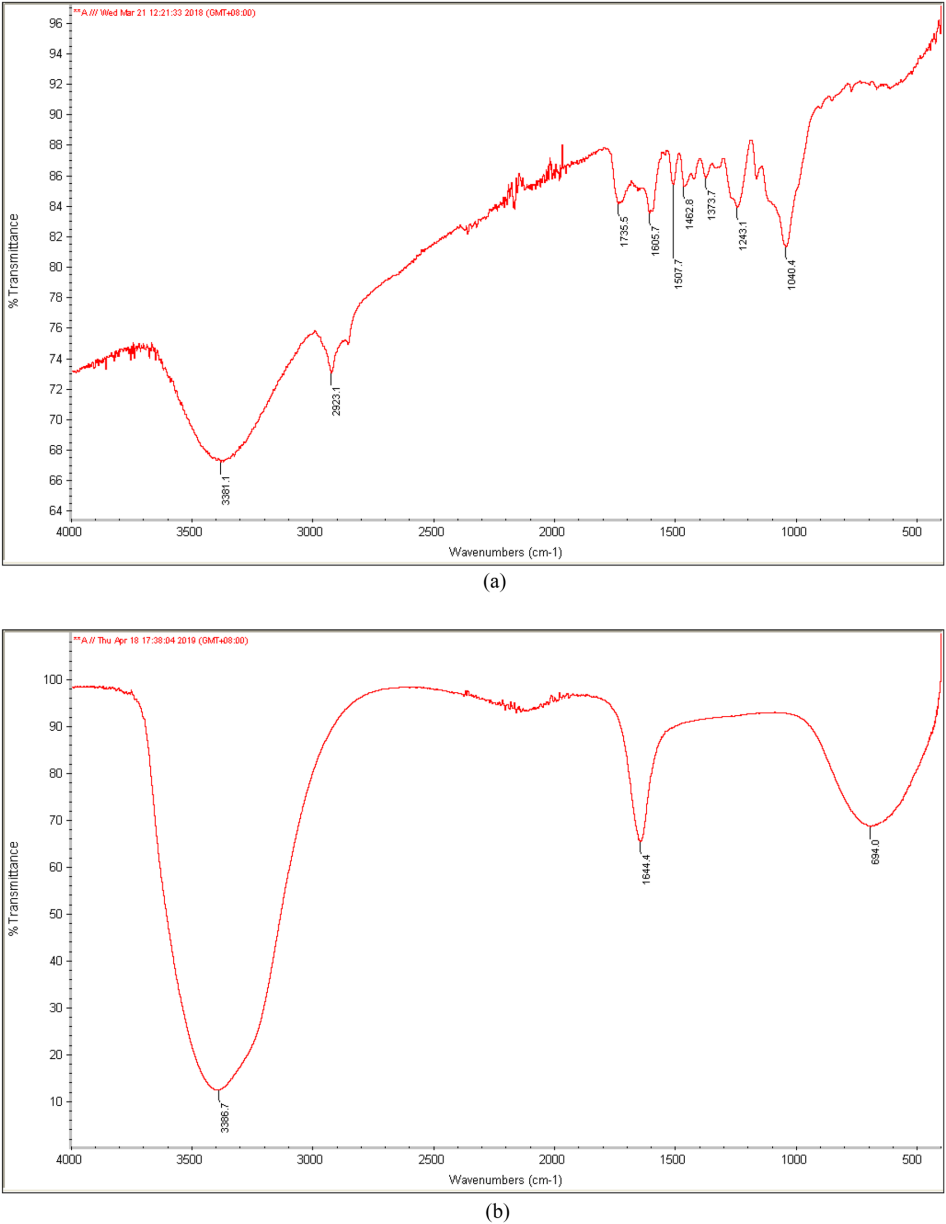

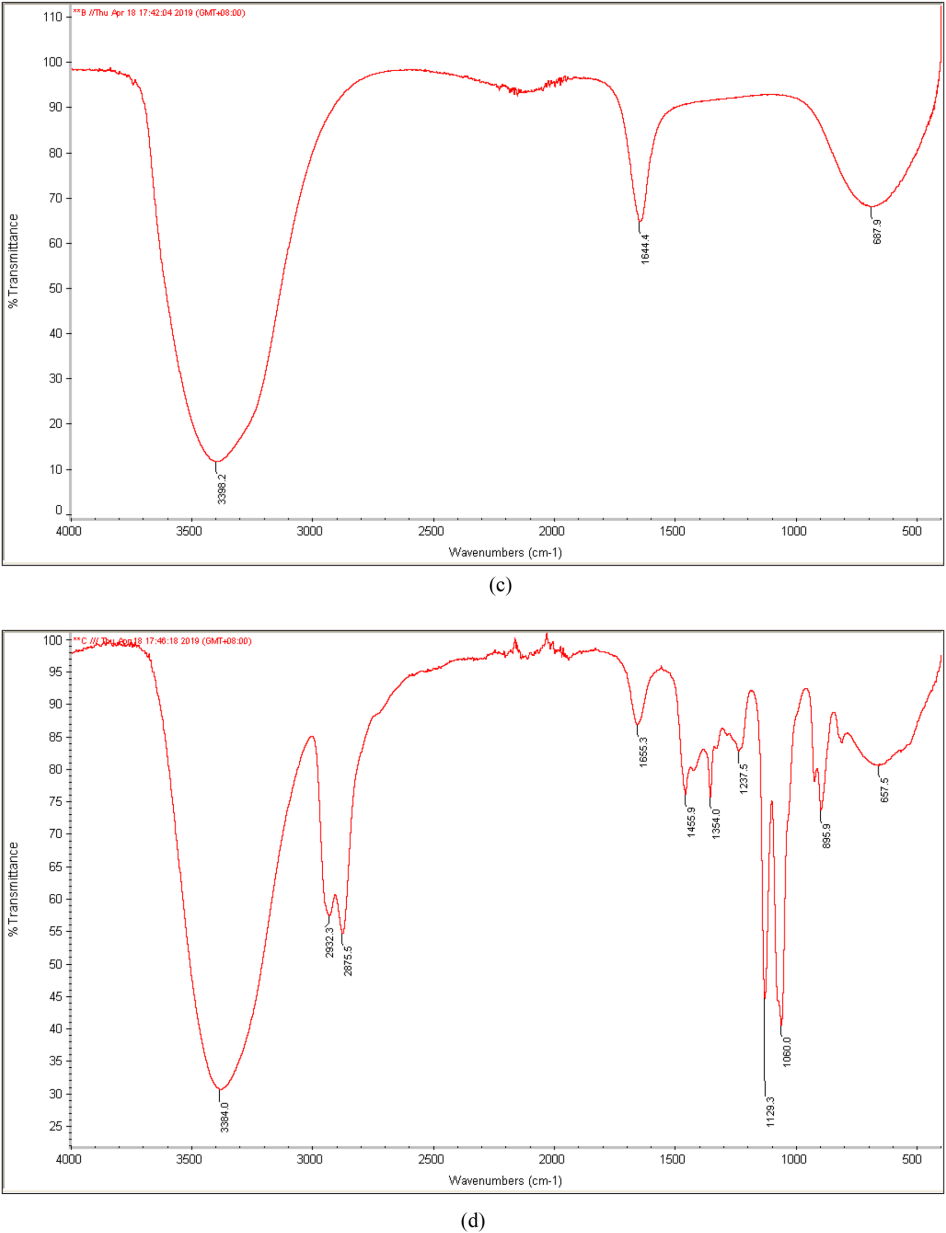
IR spectrum of (**a**) PKS, (**b**) CDs for sample A4, (**c**) CDs for sample B5, and (**d**) CDs for sample C1.

Moreover, functional groups on the surface of PKS and CDs were characterised by FTIR. Figure 2 shows the IR spectrum of PKS and CDs (sample A4, B5 and C1). For IR spectrum of PKS, it was noted that there are O–H stretching at 3381.1 cm^−1^, C–H stretching (sp^3^) at 2923.1 cm^−1^, C=O stretching of carbonyl groups at 1735.5 cm^−1^, C=C stretching (aromatic) at 1605.7 cm^−1^ and 1507.7 cm^−1^ as well as C–O stretching at 1040.4 cm^−1^^31^. The IR spectrum of A4, B5, and C1 CDs samples displayed the similar functional groups: hydroxyls (–OH stretching) and alkenes (–C=C stretching) at a stronger absorption intensity, an indication of the successful transformation of PKS into CDs with carbon core consisting of C=C elementary unit^20^. The C1 sample was also accompanied by the appearance of new bands (C–O–C stretching) at 1129.3 cm^−1^ and 1060.0 cm^−1^ as well as C–H stretching with spectrum between 2850–3000 cm^−1^. The presence of asymmetric stretching vibrations of C–O–C could be a sign of more complete intermolecular dehydration and polymerisation that only took place at higher temperature, as reported in other literature^32^. Since DEG has higher boiling point than water, it allowed a more complete reaction for the formation of CDs.

Surprisingly, amino groups (–NH_2_) were not detected for B5 CDs, which might be a sign that chitosan did not react well with the PKS to form CDs with amino functional groups. Past literature suggested that simple molecules such as acetylacetone could react with nitrogen functional group compounds (e.g. ammonia) to form amide-rich organic precursors^32^. These amide-rich organic precursors were then hydrolyzed and carbonized to form carbon core with surface modified with a large number of nitrogen-containing functional groups. Huge and complex natural carbon precursors such as PKS in this case might react poorly with chitosan or lack of reactive functional groups for interacting with chitosan. Subsequently, the CDs formed did not show the presence of amino functional groups. Based on the IR spectrum of all sets of CDs, the functional group that contributed to characterization of CDs is oxygen-containing functional groups such as O–H, C=O and C–O–C stretching. These oxygen-containing functional groups found on the surface of the synthesised CDs most likely were derived from the oxidation of hydroxyl groups in PKS during carbonisation process, which played an important role in determining the photoluminescence intensity of CDs^20,31^. These functional groups would trap the excitons under excitation and the radiative recombination of those surface-trapped excitons. The results of FTIR were in consistent with XPS spectra, where the high resolution C1s spectrum had four characteristic peaks at the binding energy of 284.6 eV, 285.9 eV, 287.6 eV, and 288.9 eV, attributing to C–C, C–OH, C=O, and O–C=O, respectively^42,45–47^ (Supplementary document).

**Figure 3:**
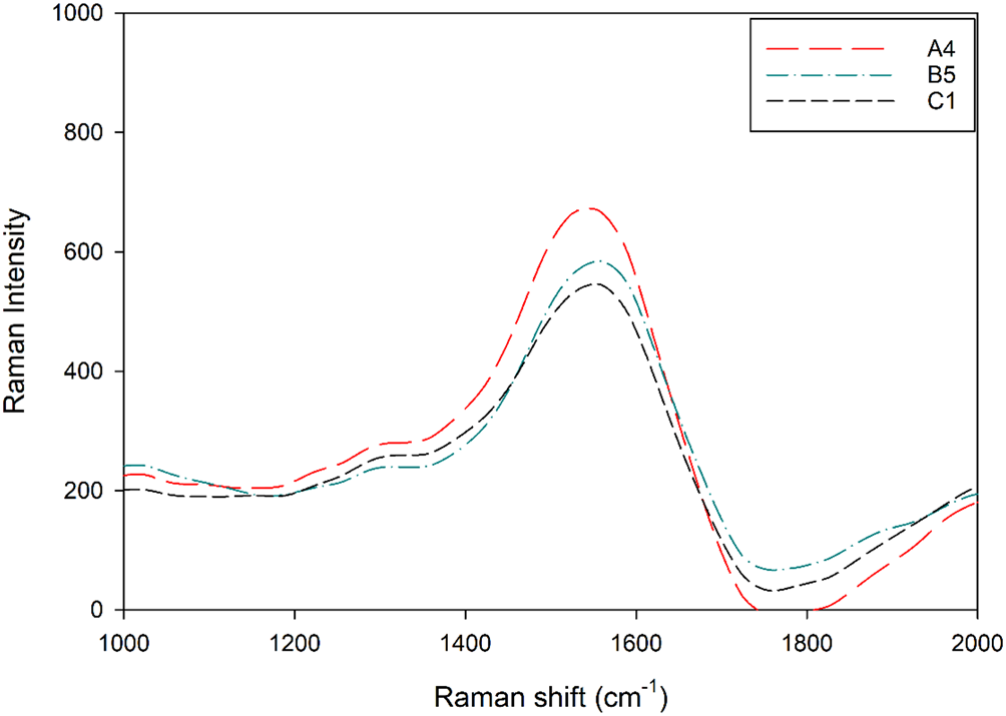
Raman spectrum of CDs.

The Raman spectrum of the CDs as shown in Fig. 3 exhibited G-band (sp^2^ hybridized) peak with high intensity at around 1580 cm^−1^ and D-band (sp^3^ hybridization) shoulder at 1330 cm^−1^^42^. The D-band could be related to the presence of sp^3^ defects while the G-band is associated with in-plane vibration of sp^2^ carbon^32,48^. The obviously low excitation at D-band could be attributed to the lack of covalent functionalization where the sp^3^ hybridization normally acts as the strong scattering centers of electrons^49^. This observation aligns with the finding reported by Zhang et al. (2018) where the lack of electron scattering center would give rise to weak D-band^50^.

### Photoluminescence properties

**Figure 4:**
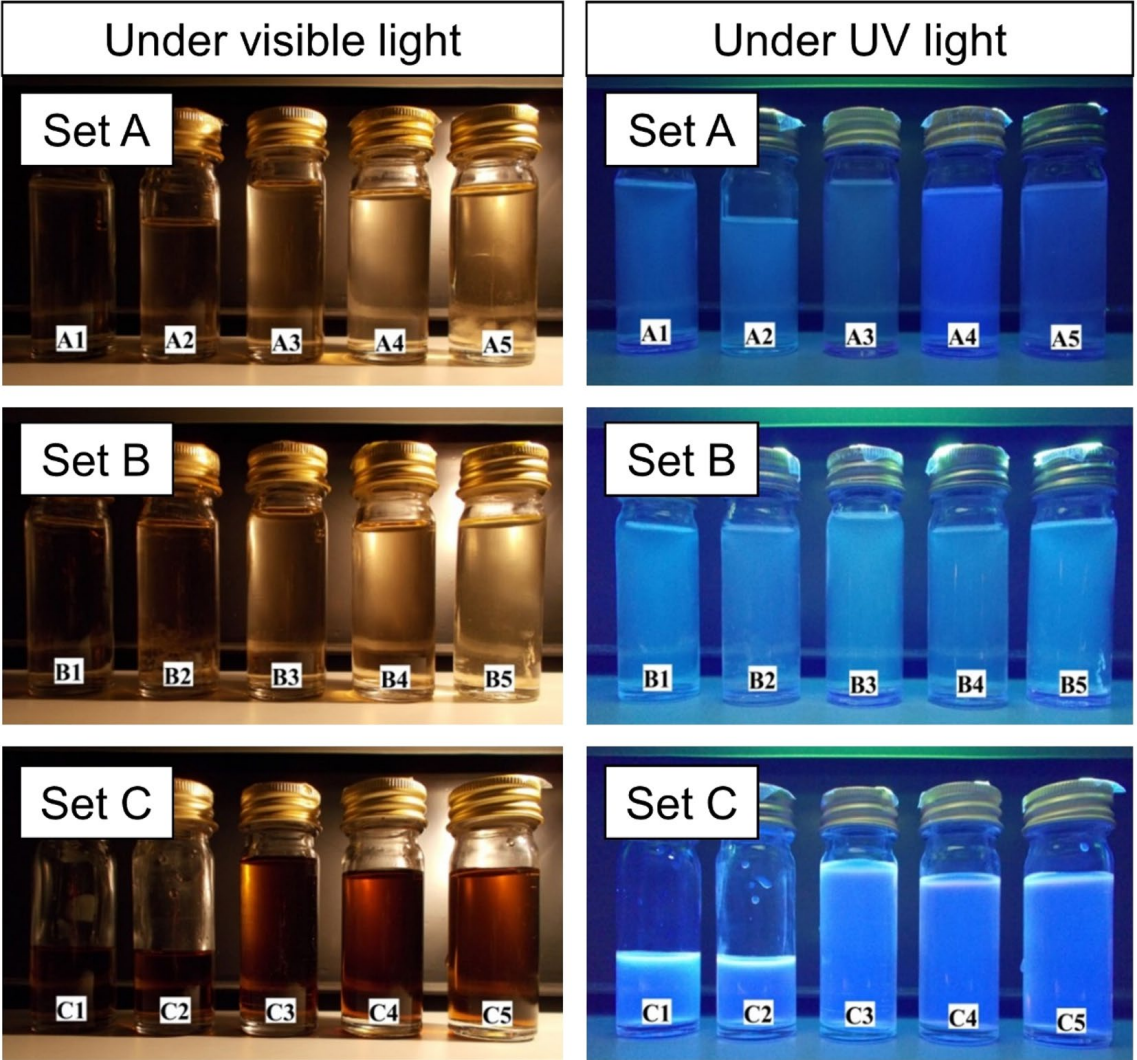
Condition of CDs solution under visible light and UV light.

The CDs solutions appeared transparent or translucent under visible light except the CDs prepared with DEG medium, which acquired light brown colour (Fig. 4). The colour of all the CDs samples remained stable for several weeks. As shown in Fig. 4, CDs from set A and set B emitted a weak blue luminescence while set C emitted a strong blue luminescence upon excitation under a 365 nm UV light. The brightness of the luminescence observed is closely linked to the photoluminescence properties of the CDs, which would be further elaborated below.

**Figure 5:**
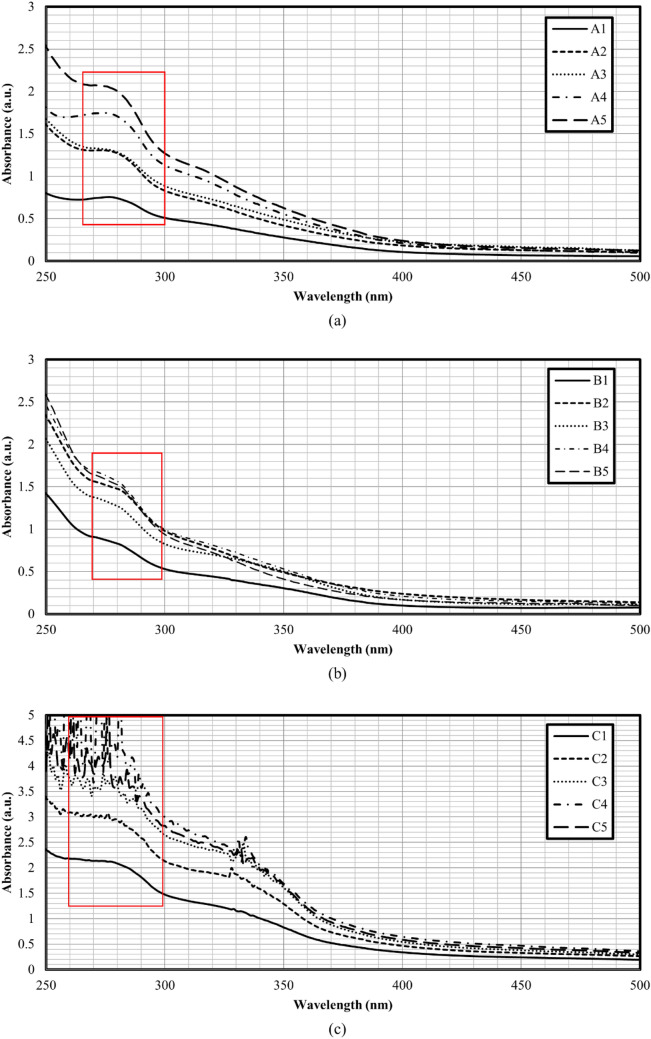
UV–Vis absorption spectrum of (**a**) Set A, (**b**) Set B, and (**c**) Set C.

The UV–Vis absorption spectra of all sets of CDs were presented in Fig. 5. Based on all the UV–Vis absorption spectra obtained, strong absorption peak at 280 nm along with a shoulder around 300 nm were observed. The peak at 280 nm was due to blue-shifted π − π^*^ transition of the conjugated C=C groups from the carbon core^31^. Meanwhile, the shoulder was attributed to the n − π^*^ transition of C=O groups (typical characteristic of fluorescent CDs) found on the surface of CDs^31,32^. According to the trends of UV–Vis absorption spectra, it can be said that the absorption intensity for set A and B increased when heating period was prolonged. This could be an indication that more carbon cores were generated with the increase of reaction time. Such postulation was supported with the finding reported by He et al. (2017) and aligned well with the optimal quantum yield for set A and B CDs (more CDs indicate higher quantum yield) at longer heating duration^31,32^. For CDs synthesised with DEG as the reacting medium (Fig. 5c), the absorbance intensity also increased with the heating duration. However, further heating the CDs for set C has probably damaged the surface (Set C3–C5 possessed quantum yield less than 6%) and resulted in the loss of capability responding to light.

**Figure 6:**
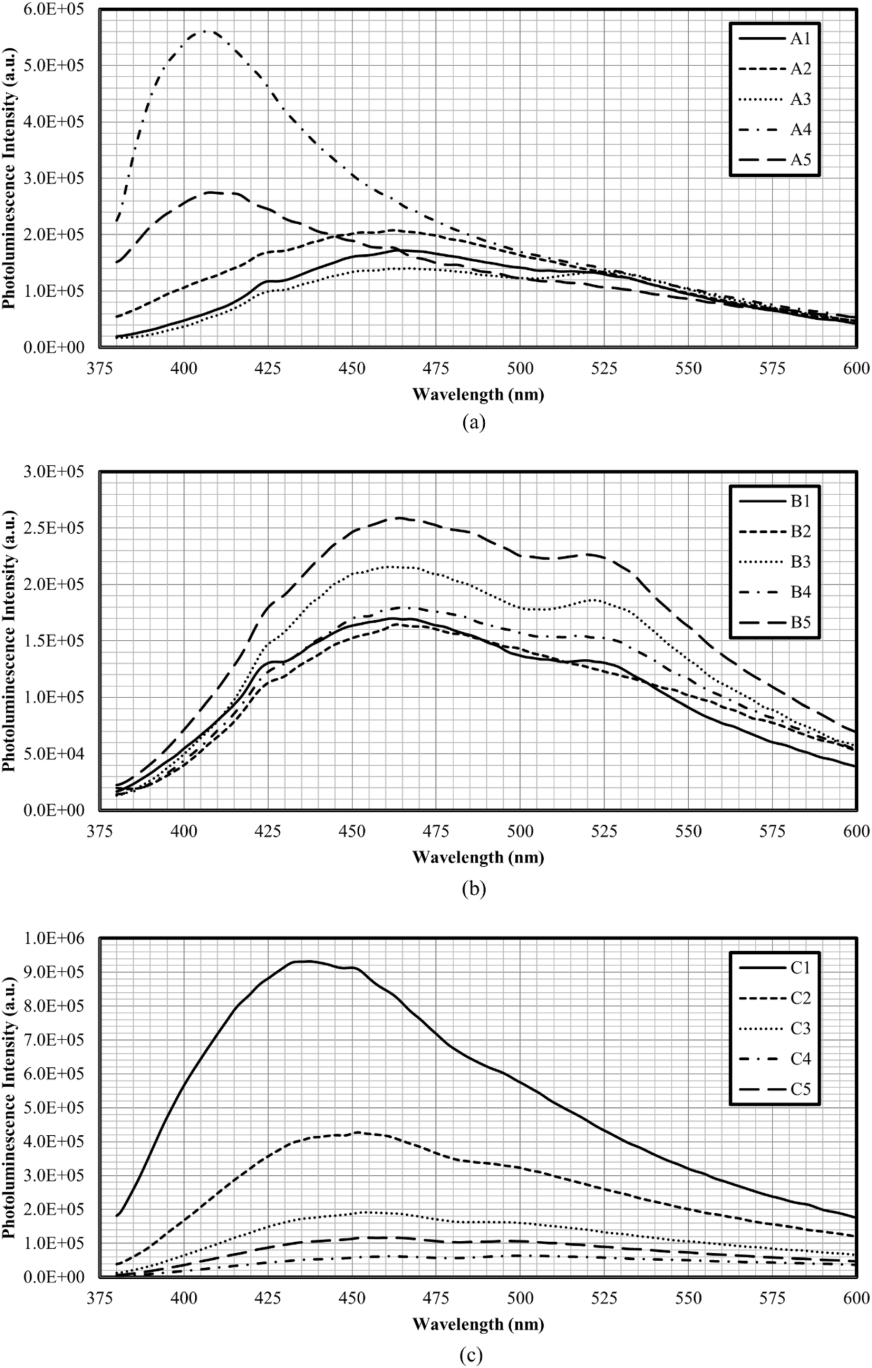
Photoluminescence spectrum (excited at 370 nm) of (**a**) Set A, (**b**) Set B, and (**c**) Set C.

Figure 6 shows the photoluminescence spectra of CDs for set A, B, and C, respectively, at the excitation wavelength of 370 nm. Based on the photoluminescence spectra obtained, CDs achieved the highest photoluminescence intensity at heating periods of 4 min, 5 min, and 1 min for set A, B, and C, respectively. According to the previous studies, photoluminescence intensity increased when the microwave irradiation periods increased (generation of more CDs)^24^. But the photoluminescence intensity will decrease at certain heating periods when turn-off phenomenon occurs. Over the optimal heating period, the CDs formed would experience surface damage that reduces the photoluminescence intensity. However, for the CDs synthesised with DEG, the highest photoluminescence intensity was achieved at the shortest heating duration. This could be due to the higher boiling point of DEG that facilitated fast heating for the formation of CDs, and further heating would lead to the damage of CDs surface (as verified by the decrease of quantum yield from 44.0% at 1 min to 3.9% at 5 min of microwave irradiation duration). Table 3 presents the emission peak of all sets of synthesised CDs upon excitation at 370 nm. The emission peaks of Set C CDs exhibited moderate redshift from 438 to 459 nm with the increase of heating period^32^. This phenomenon was probably due to the variation of CDs sizes and the surface states^29^.

**Table 3:** The emission peak of all the synthesised CDs (excited at 370 nm).

Parameter		Heating period (min)				
		1	2	3	4	5
Emission peak (nm)	A	466	462	466	407	410
	B	464	467	463	467	465
	C	438	452	456	462	459

### Cellular imaging

**Figure 7:**
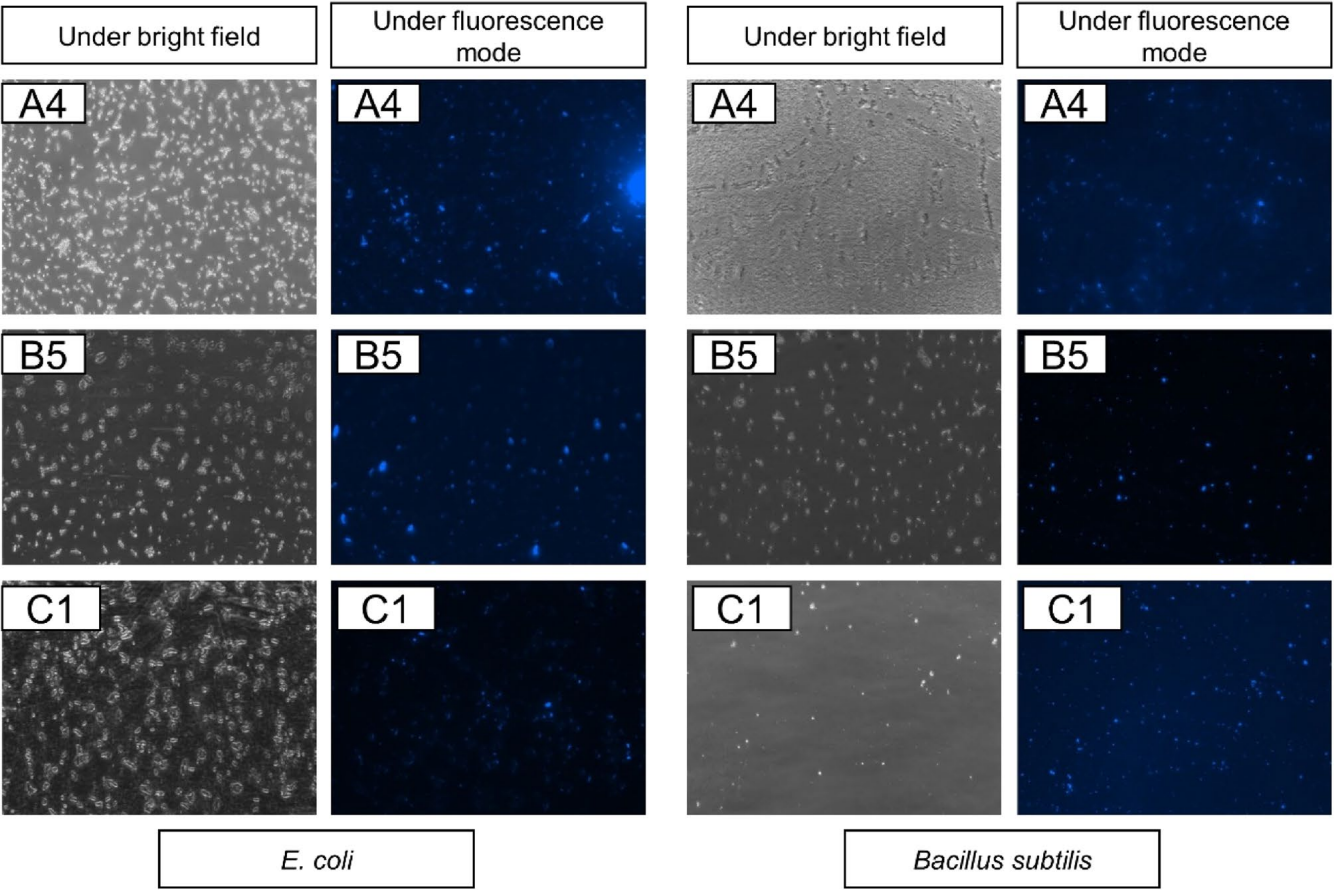
Fluorescence microscope images under bright field and fluorescence mode (magnification 20 ×).

Figure 7 presents the fluorescence microscope images on both of the bacterial cells under different exciter filters which are bright field and fluorescence mode at excitation of 340–380 nm and emission of 435–485 nm. The fluorescence microscopy images showed that quite some fluorescent bacterial cells were observed. These images revealed that CDs could be effectively attached to both of the bacterial cells. The mechanism involved in this case can be related to the tiny spherical CDs prepared from PKS and hydroxyl groups existed on the surface of the CDs, which contributed to the binding of CDs to the bacterial cells through the amine groups (such as peptides, proteins, and amino acids) present on the bacterial surface^41^.

### Detection of heavy metal ions

**Figure 8:**
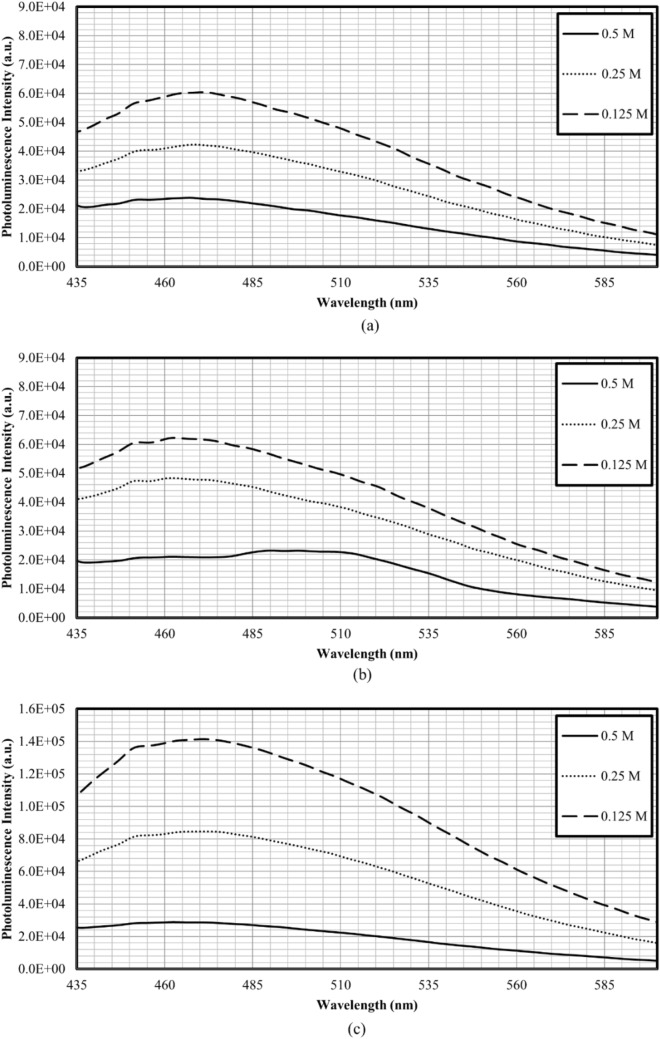
Photoluminescence spectrum on different concentration of copper (II) ions (excited at 370 nm) of (**a**) A4, (**b**) B5, and (**c**) C1.

**Figure 9:**
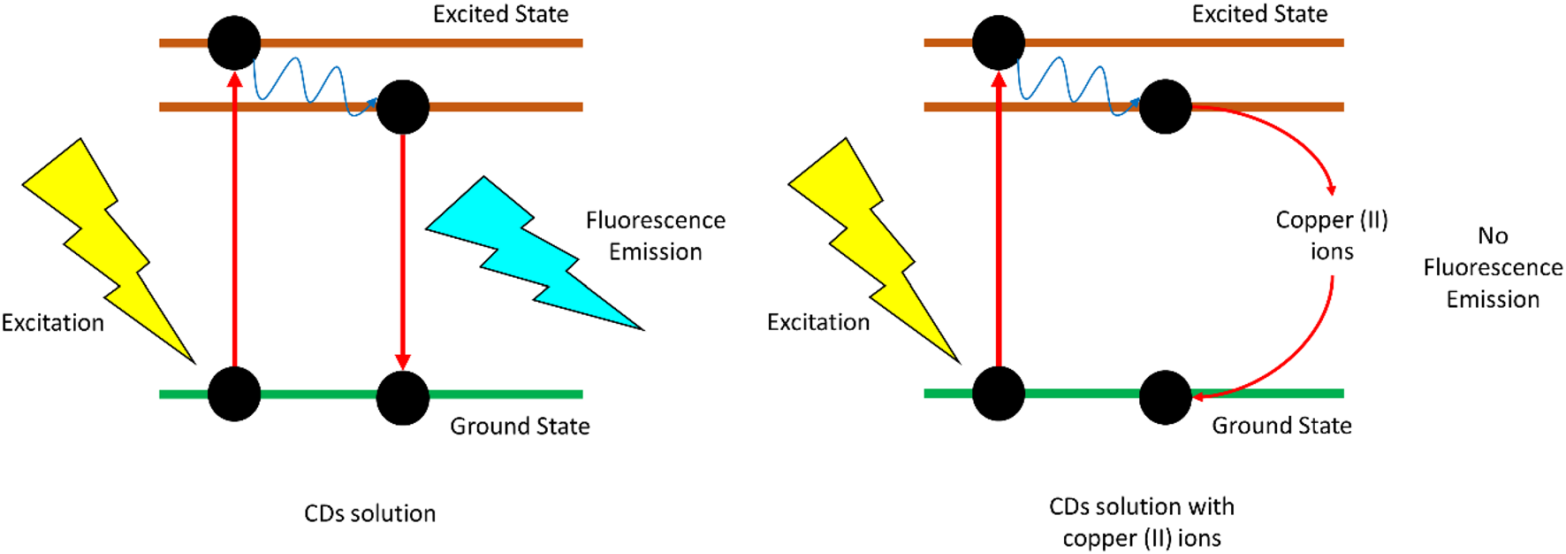
Proposed fluorescence quenching mechanism for CDs by copper (II) ions.

Potential of CDs as heavy metal ions (copper (II) ions) sensing probe has been evaluated by observing the changes in photoluminescence spectrum with the addition of metal ions. Figure 8 show the effect of copper (II) ions concentration on the emission spectra of solution dosed with A4, B5, and C1 CDs, respectively. It can be seen that the photoluminescence intensity of the solutions decreased with the increase of copper (II) ions concentration from 0.125 to 0.5 M, which is an indication confirming the potential of the synthesised CDs for metal ions sensing. The fluorescence of CDs could be related to the oxygeneous functional groups such as hydroxyl and carboxylate groups (as discussed previously) present on the CDs surfaces. These functional groups are excellent chelating ligands for metal ion complexation^45,47^. The complexation between CDs and metal ions would lead to fluorescence quenching due to the formation of non-fluorescence complex between the surface functional groups of CDs and copper (II) ions^42,47^. The fluorescence quenching could be attributed to the electron/hole recombination annihilation through an efficient and reversible electron/hole transfer process^23,46^. As illustrated in Fig. 9, the photoluminescent emission of CDs which originates from the radiative recombination of excitons could be quenched by copper (II) via nonradiative electron-transfer from CDs to copper (II) ions^34^. This electron transfer pathway did not emit fluorescence and support the quenching phenomena when copper (II) ions were added to CDs solution. The degree of disturbances on the fluorescence of CDs increased with the presence of more metal ions, where it could be clearly seen that the photoluminescence intensity of all sets of CDs flattened when the concentration of copper (II) ions reached 0.5 M. The quenching efficiency for A4, B5, and C1 varied between 89–95%, 75–91%, and 85–97%, respectively. The slightly higher quenching efficiency for C1 could be attributed to its higher quantum yield that provided more oxygen-containing functional groups to interact with the metal ions.

### Removal of heavy metal ions

**Figure 10:**
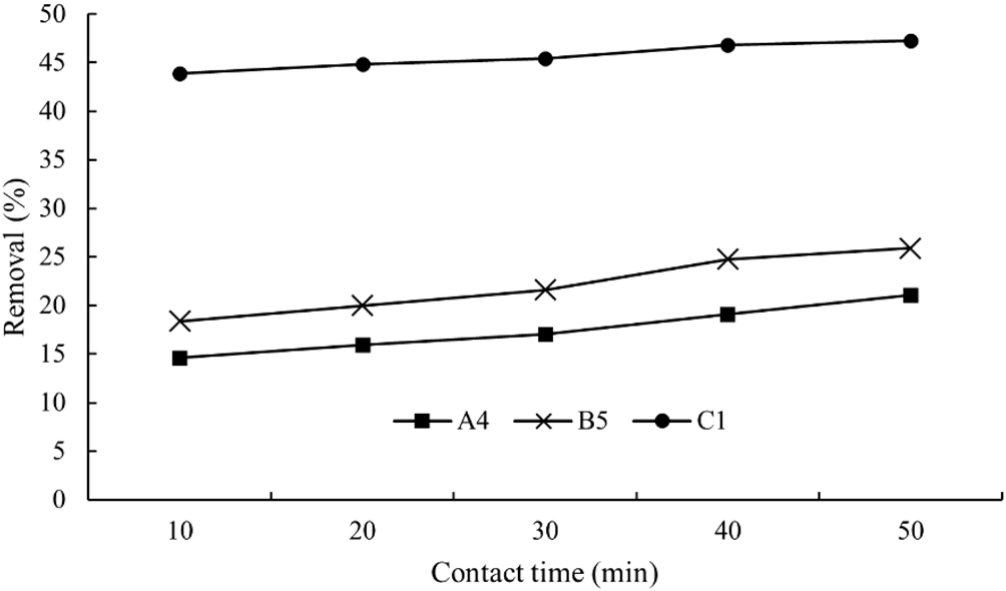
Removal of heavy metal ions with respect to contact time.

**Figure 11:**
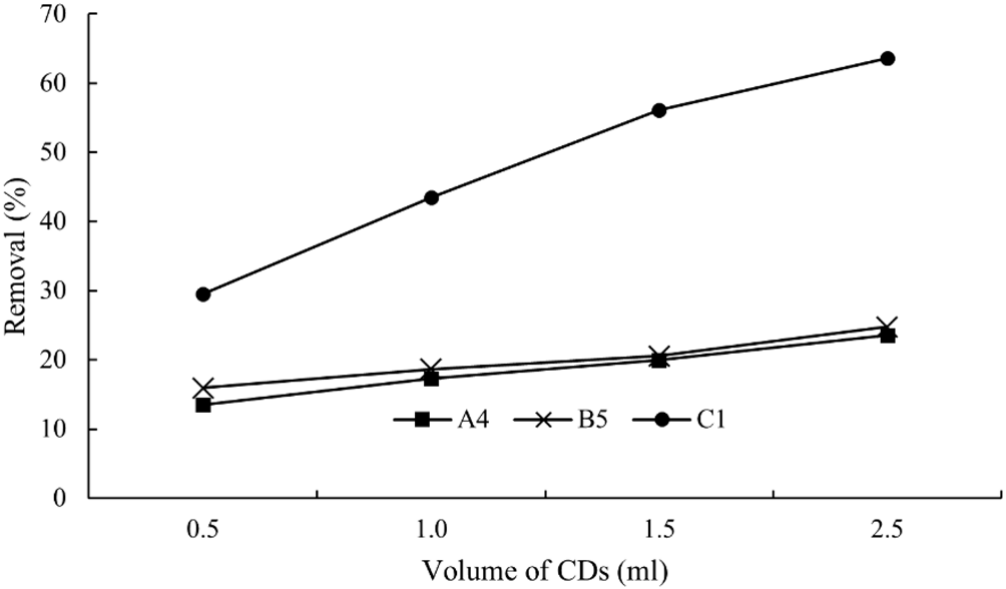
Removal of heavy metal ions after 50 min with respect to the volume of CDs solution.

Figures 10 and 11 show the removal efficiencies of copper (II) ions under various contact times and CDs dosages. Obviously, C1 CDs outperformed A4 and B5 in terms of removal rate and efficiency. C1 CDs took 10 min to obtain 47% removal efficiency while A4 and B5 CDs failed to attain this removal efficiency even after 50 min. In another case, 2.5 ml of C1 CDs could remove 73% of copper (II) ions, showing higher removal capacity as compared to A4 and B5 (27% removal) at the similar dosage. Both these observations could be attributed to the higher quantum yield of C1 CDs with more functional groups that resulted in more CDs with functional groups to interact with the copper (II) ions. The removal process of heavy metal ions probably took place through the adsorption mechanism by electrostatic interaction between the CDs and copper (II) ions. The negatively charged hydroxyl groups on CDs could interact with the positively charged copper (II) through electrostatic interaction and subsequently led to the removal of the metal ions^51^.

## Conclusions

In summary, it can be concluded that PKS, a biomass waste from the palm oil industry, can be the low-cost and easily available precursor for the synthesis of CDs via microwave irradiation method. It was discovered that DEG being a reacting medium with high boiling point can facilitate the fast and more complete formation of CDs (shorter duration) from PKS as compared to water as the reacting medium. This was translated to the production of CDs with higher quantum yield (44.0% vs 26.3%) which displayed better absorbance and photoluminescent intensities. Surprisingly, the presence of chitosan did not improve the quantum yield of CDs of set B, which could be a sign that chitosan did not interact with complex carbon precursors such as PKS to form CDs with amino functional groups. Nonetheless, all the synthesised CDs have been successfully used in biological cell imaging, a sign that shows the CDs could be nontoxic to living bacteria. On top of that, the CDs could be used to detect copper (II) ions through fluorescence quenching. The higher quantum yield of CDs synthesised from DEG reacting medium has provided it a greater adsorption capability as shown by its better and faster removal of copper (II) ions. Overall, the CDs synthesised from PKS possessed the potential to be used for bioimaging and detection of pollutants.

## Supplementary information

Supplementary Information 1.
