# Supplementary material for: Microwave-assisted conversion of palm kernel shell biomass waste to photoluminescent carbon dots
Source: Sci Rep. 2020 Dec 3;10:21199. doi: 10.1038/s41598-020-78322-1 (PMC7712893; doi:10.1038/s41598-020-78322-1)
Supplement: Supplementary file 1 — Supplementary Information 1. [file 41598_2020_78322_MOESM1_ESM.docx]

**Supplementary Document**

**Microwave-assisted Conversion of Palm Kernel Shell Biomass Waste to Photoluminescent Carbon Dots**

Wei Lun Ang^1,2^*, Cheldclos A/L Boon Mee^1^, Nonni Soraya Sambudi^3,4^, Abdul Wahab Mohammad^1,2^, Choe Peng Leo^5^_,_ Ebrahim Mahmoudi^1,2^, Muneer Ba-Abbad^6^, Abdelbaki Benamor^6^

^1^Department of Chemical and Process Engineering, Faculty of Engineering & Built Environment,

Universiti Kebangsaan Malaysia, 43600 Bangi, Selangor Darul Ehsan, Malaysia.

^2^Centre for Sustainable Process Technology (CESPRO), Faculty of Engineering & Built Environment,

Universiti Kebangsaan Malaysia, 43600 Bangi, Selangor Darul Ehsan, Malaysia.

^3^Chemical Engineering Department, Universiti Teknologi PETRONAS, 32610 Seri Iskandar, Perak Darul Ridzuan, Malaysia.

^4^Center for Advanced Integrated Membrane System (AIMS), Universiti Teknologi PETRONAS, 32610 Seri Iskandar, Perak Darul Ridzuan, Malaysia.

^5^School of Chemical Engineering, Engineering Campus, Universiti Sains Malaysia, 14300 Nibong Tebal, Penang, Malaysia.

6Gas Processing Centre, Qatar University, P.O. Box 2713, Doha, Qatar.

*Corresponding author: [wl_ang@ukm.edu.my](mailto:wl_ang@ukm.edu.my)

XPS C1s Spectra for CDs sample


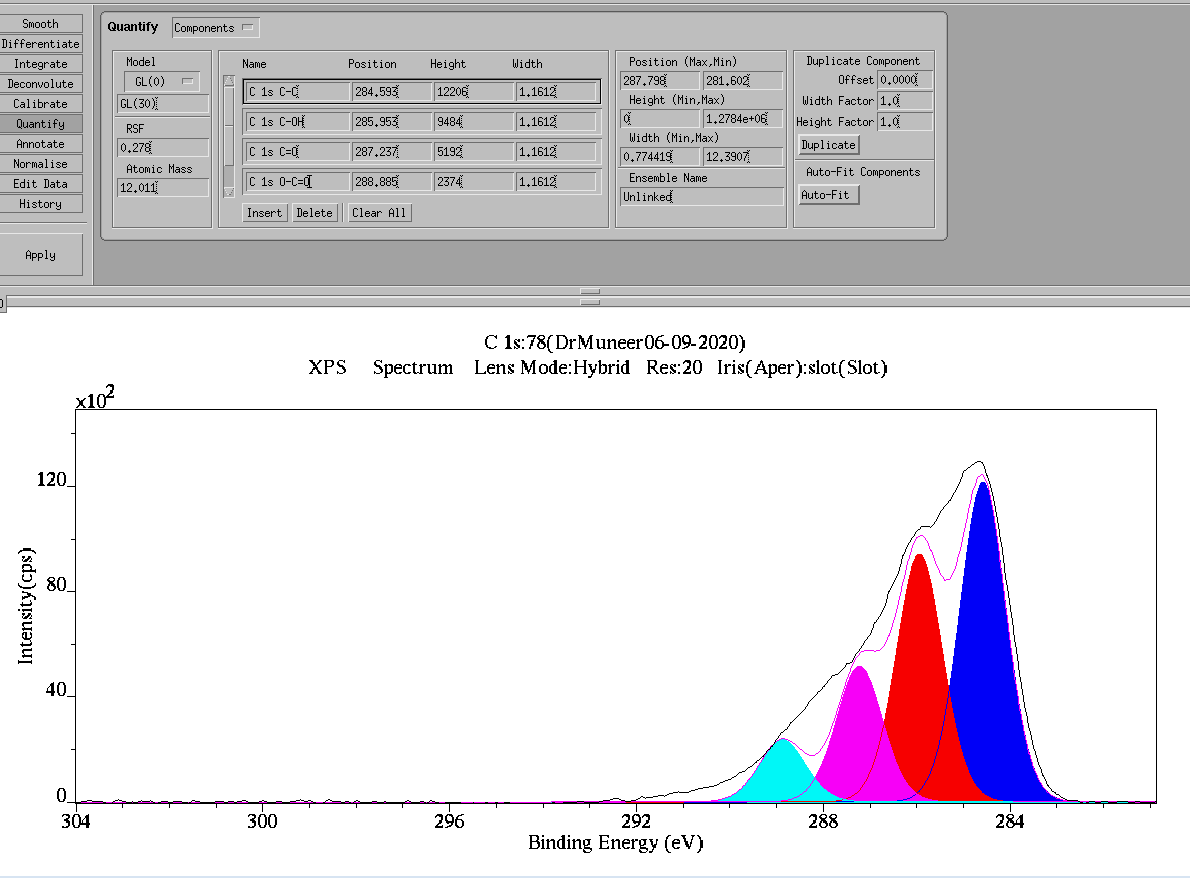


| Peak | Type | Position BE (eV) | FWHM (eV) | Raw Height (cps) | RSF | Atomic Mass | Atomic Conc % | Mass Conc % |
| --- | --- | --- | --- | --- | --- | --- | --- | --- |
| C is C-C | Comp | 284.593 | 1.161 | 12205.9 | 0.278 | 12.011 | 41.73 | 41.73 |
| C is C-OH | Comp | 285.953 | 1.161 | 9483.7 | 0.278 | 12.011 | 32.42 | 32.42 |
| C is C=O | Comp | 287.237 | 1.161 | 5192.2 | 0.278 | 12.011 | 17.74 | 17.74 |
| C is O-C=O | Comp | 288.885 | 1.161 | 2373.5 | 0.278 | 12.011 | 8.11 | 8.11 |

Sample A4


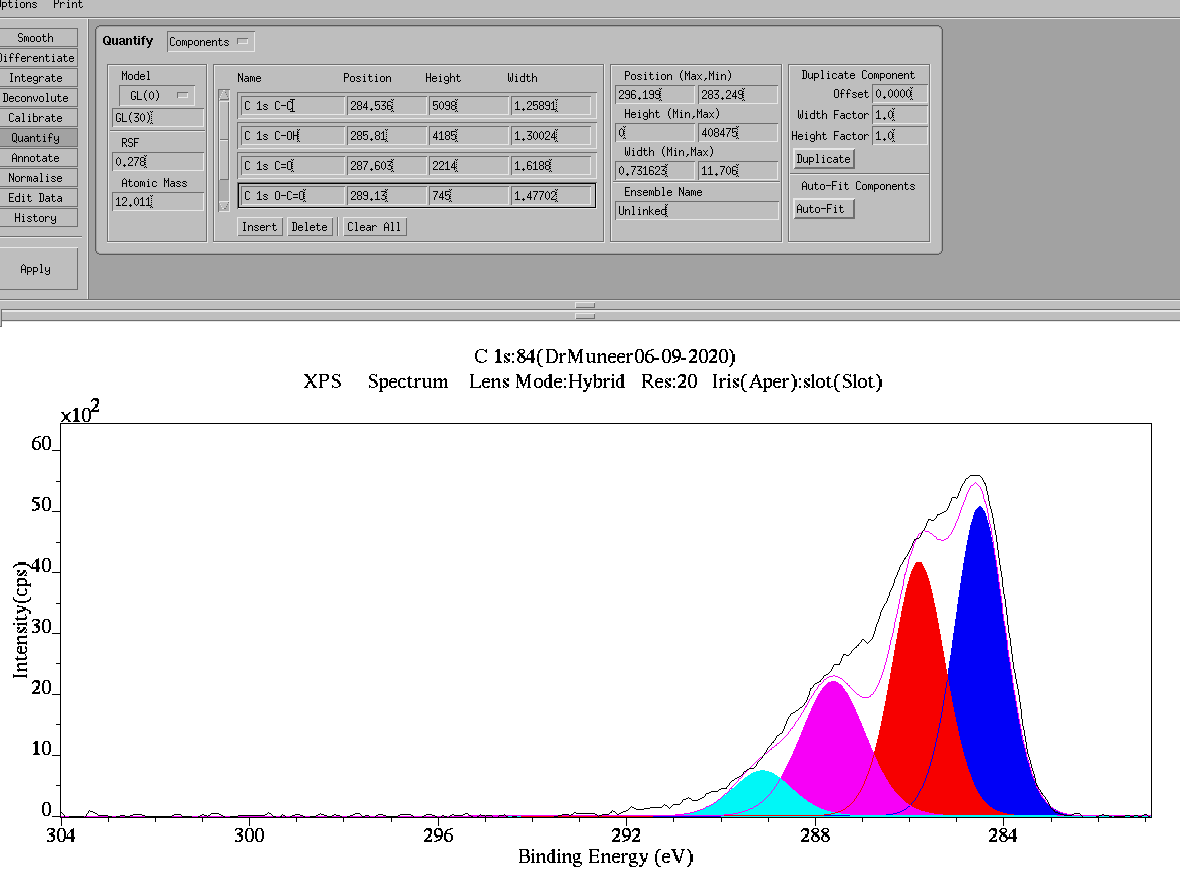


| Peak | Type | Position BE (eV) | FWHM (eV) | Raw Height (cps) | RSF | Atomic Mass | Atomic Conc % | Mass Conc % |
| --- | --- | --- | --- | --- | --- | --- | --- | --- |
| C is C-C | Comp | 284.536 | 1.259 | 5098.0 | 0.278 | 12.011 | 41.65 | 41.65 |
| C is C-OH | Comp | 285.810 | 1.300 | 4184.6 | 0.278 | 12.011 | 34.18 | 34.18 |
| C is C=O | Comp | 287.603 | 1.619 | 2214.2 | 0.278 | 12.011 | 18.08 | 18.08 |
| C is O-C=O | Comp | 289.130 | 1.477 | 745 | 0.278 | 12.011 | 6.08 | 6.08 |

Sample B5


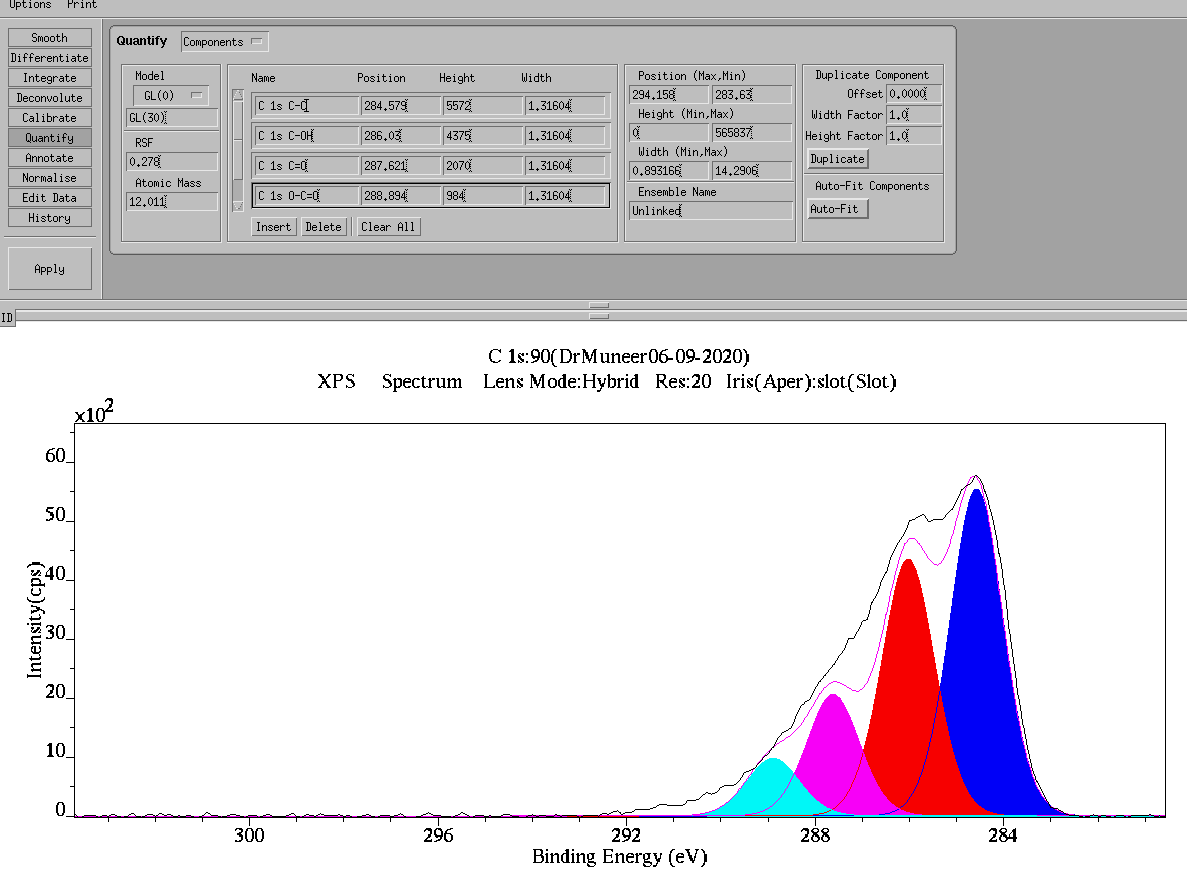


| Peak | Type | Position BE (eV) | FWHM (eV) | Raw Height (cps) | RSF | Atomic Mass | Atomic Conc % | Mass Conc % |
| --- | --- | --- | --- | --- | --- | --- | --- | --- |
| C is C-C | Comp | 284.579 | 1.316 | 5572.0 | 0.278 | 12.011 | 42.87 | 42.87 |
| C is C-OH | Comp | 286.030 | 1.316 | 4375.0 | 0.278 | 12.011 | 33.65 | 33.65 |
| C is C=O | Comp | 287.621 | 1.316 | 2070.0 | 0.278 | 12.011 | 15.92 | 15.92 |
| C is O-C=O | Comp | 288.894 | 1.316 | 984.0 | 0.278 | 12.011 | 7.57 | 7.57 |

Sample C1
